# Hypertension and Dementia: Epidemiological and Experimental Evidence Revealing a Detrimental Relationship

**DOI:** 10.3390/ijms17030347

**Published:** 2016-03-08

**Authors:** Marialuisa Perrotta, Giuseppe Lembo, Daniela Carnevale

**Affiliations:** 1 Department of Angiocardioneurology and Translational Medicine, IRCCS Neuromed, 86077 Pozzilli, Italy; m.perrotta1@studenti.unimol.it (M.P.); daniela.carnevale@neuromed.it (D.C.); 2 Department of Molecular Medicine, “Sapienza” University of Rome, 00161 Rome, Italy

**Keywords:** hypertension, cerebrovascular dysfunction, vascular cognitive impairment, molecular mechanisms

## Abstract

Hypertension and dementia represent two major public health challenges worldwide, notably in the elderly population. Although these two conditions have classically been recognized as two distinct diseases, mounting epidemiological, clinical and experimental evidence suggest that hypertension and dementia are strictly intertwined. Here, we briefly report how hypertension profoundly affects brain homeostasis, both at the structural and functional level. Chronic high blood pressure modifies the cerebral vasculature, increasing the risk of Aβ clearance impairment. The latter, excluding genetic etiologies, is considered one of the main causes of Aβ deposition in the brain. Studies have shown that hypertension induces cerebral arterial stiffening and microvascular dysfunction, thus contributing to dementia pathophysiology. This review examines the existing and the updated literature which has attempted to explain and clarify the relationship between hypertension and dementia at the pathophysiological level.

## 1. Introduction

Hypertension is considered one of the leading causes of morbidity and mortality worldwide, despite considerable success of treatment and prevention [1]. Although often neglected, the brain is one of the main target organs subjected to detrimental dysfunctions caused by high blood pressure. Indeed, hypertension is the leading risk factor for acute cerebrovascular events like stroke [2], but it is also known to be the cause for chronic disorders severely affecting cognitive function, *i.e.*, vascular cognitive impairment (VCI) as well as dementia [3]. Although the typical neuropathological features of Alzheimer’s disease (AD)—and more in general of dementia—have been classically ascribed to alterations in the neuronal components of the brain [4], it is now well known that late-life dementia is frequently attributable to a vascular etiology [5]. On this issue, hypertension is one of the most powerful vascular risk factors increasingly associated with the development of late-life dementia.

Hypertension has deep effects on brain vessels: it alters the structure of cerebral vasculature and causes atherosclerotic plaque development, both in cerebral arteries and arterioles [6]. Moreover, hypertension is also responsible for the alteration of all the functional adaptive mechanisms of brain vessels, e.g., resting cerebral blood flow (CBF), functional hyperemia and endothelium-dependent relaxation of cerebral blood vessels [7,8]. More importantly, it has been clearly reported how hypertension can alter cerebrovascular autoregulation mechanisms, leading to a right shift of the pressure-flow curve [9]. Thus, in hypertension, higher perfusion pressures are needed to maintain constant the CBF levels.

The association between hypertension and dementia is supported by a great clinical relevance. In the last decades, there have been various basic science research reports attempting to investigate the specific molecular link between the two pathological conditions. However, the genetic mouse models of AD could not reproduce the manifold aspects of the human neuropathology that, in the vast majority of patients, contributes to late-life dementia.

In this scenario, our group has developed a murine model of hypertension-induced AD, attempting to dissect the molecular mechanisms at the basis of the relationship between chronic hypertension and dementia-like features [10,11,12]. More recently, other studies have further supported this novel perspective in approaching late-life dementia [13,14,15]. Several epidemiological data have associated, in the elderly, increased blood pressure, angina and arterial fibrillation with a more rapid decline of cognitive functions [16,17,18,19]. An epidemiological study reported that the treatment of vascular risk factors slows the cognitive decline in AD patients [18]. This evidence suggests the importance of detecting and controlling increased blood pressure in midlife.

Here we will briefly review the main research describing the structural and functional alterations induced by chronic hypertension in the brain. Then, we will examine basic science papers that have attempted to unveil the pathophysiological link between hypertension and dementia.

## 2. The Hypertensive Response in the Brain: Structural and Functional Alterations in Cerebral Vasculature

Hypertension, defined as a chronic elevation in blood pressure exceeding 140 mmHg systolic or 90 mmHg diastolic, can lead to target organs damage, such as brain, heart and kidneys, inducing detrimental events. Despite the large number of existing treatments to lower blood pressure, the mechanisms leading to organ damage as a consequence of blood pressure raising is still unknown [20].

Hypertension primarily affects the brain [21], leading to cognitive disorders and AD [22]. Besides the most commonly recognized acute effects like stroke [21], in the brain blood pressure increase leads to a breakdown of cerebral vasculature and, in turn, causes disruption of neurovascular units. Moreover, the lack of regulation at this level induces a degeneration of the cerebral tissues and, consequently, brain damage. Many studies have focused on how hypertension may disrupt brain homeostasis [23]: indeed, the comprehension of such mechanisms could be important for the development of selective therapeutic strategies. These new findings may provide a unique opportunity for the dissection of the cerebrovascular effects of hypertension.

One of the devastating effects exerted by hypertension on the brain is the development of atherosclerotic plaques, which alter the cerebral blood vessels structure [21]. Atherosclerosis plaque can release fragments causing cerebrovascular occlusions and ischemic injury [24,25].

On the other side, small arterioles occlusion in the subcortical regions can affect multiple cognitive domains—such as those related to executive and cognitive functions [24]—thus increasing the risk of VCI.

Moreover, the chronic mechanical stress induced by hypertension in the brain, could generate a condition known as small vessel disease (SVD) [26,27]. SVD is a cerebral microvascular pathology probably caused by structural alterations in small arteries, arterioles and capillaries supplying the cortical white matter and basal ganglia [14]. Hypertension is hence considered the main pathogenic factor for SVD, responsible for lacunar infarcts, white matter lesions (leukoaraiosis) and microinfarcts [28,29]. Currently, SVD represents a major cause of VCI, contributing up to 45% of dementia cases [28,29].

Hypertension triggers the adaptive remodeling of cerebral arteries leading to a modification of smooth muscle cells. On the one side, smooth muscle cells start to proliferate and to increase their size invading the lumen of cerebral arteries. This phenomenon is widely known as the hypertrophic remodeling induced by hypertensive challenges and resulting in an increased wall thickness in conjunction with a reduction of lumen vessels. On the other side, the eutrophic remodeling of cerebral arteries leads to a reduction of vessels lumen without changes in wall thickness [30]. The vascular wall undergoes structural changes depending on different kinds of stimuli. In response to hypertensive stimuli, vessels wall collagen increases, promoting a process called vascular stiffening [31,32].

A reasonable amount of evidence shows that the reduction in peripheral resistance in the individual patient correlates with an improvement in abnormal remodeling of resistance vessel [33]. Hypertension worsens vascular dysfunctions, a typical condition of ageing, and accelerates the same processes that contributed to the development of hypertension itself, such as endothelial dysfunction, vascular remodeling and arterial stiffness [34]. For example, endothelial dysfunction, considered the earliest event in the onset of vascular disease, is able to reduce the resting blood flow, causing hypoperfusion in the brain [35].

Besides the so far described structural modifications, hypertension is also able to alter all the functional adaptive mechanisms of brain vessels. In fact, it has been reported that hypertensive patients suffer from alterations in resting CBF, reflecting an increase in cerebrovascular resistance to counteract the increase in perfusion pressure and to keep CBF distribution in tissues constant [36,37]. A reduction of CBF can also affect brain activity and vascular tone, accelerating the cerebrovascular effects caused by aging [38]. Increased blood pressure alters the hyperemia, which is considered a functional mechanism able to maintain homeostasis during brain activity. Indeed, a hypertensive stimulus can disrupt this balance causing functional damage in neurons and glia [39]. CBF is strictly related to brain activity and results attenuated in patients with chronic hypertension [26]. In experimental models of hypertension, mice with chronic angiotensin II (AngII) infusion show a marked attenuation in CBF increase induced by activation of the somatosensory cortex, which is obtained by facial whiskers stimulation [7]. To Summarize, all the hypertension-induced structural alterations of cerebral blood vessels are intertwined with the functional alterations such as modifications of hyperemia, autoregulation and endothelial function. The outcome is a reduced compensatory capacity of the cerebral circulation and an increased susceptibility of the brain to vascular insufficiency, thus explaining how chronic conditions of high blood pressure levels may strongly affect cognitive functions, determining an evolution toward dementia [26,40].

## 3. Alterations of Aβ Production and BBB Functions Leading to Cerebrovascular Dysfunction in Dementia

**Figure 1:**
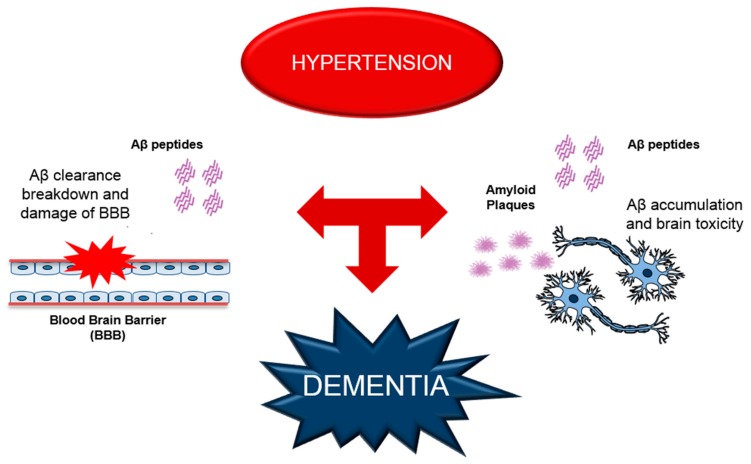
Schematic representation of hypertension-induced alterations, leading to dementia. Hypertension negatively affects the mechanisms of Aβ clearance causing a damage of BBB (blood brain barrier) and increase of Aβ accumulation in the brain, aggravating the synaptic dysfunction and the brain toxically. All these effects contribute to the deterioration of cognitive functions.

Vascular factors are the leading causes of cerebrovascular dysfunctions and hypertension represents the main challenge for the onset and progression of dementia [10,40]. AD is the best characterized chronic neurodegenerative disorder. Several studies have shown that hypertension-induced lesions and AD may have an additive or synergistic effect and produce a more severe cognitive impairment than either process alone [41,42,43], as shown in Figure 1. AD has been classically defined by the presence of typical pathological hallmarks [44]. In particular, soluble proteins’ accumulation in the perivascular space leads to the formation of insoluble complicated structures as β-sheets [45,46]. Many secreted, circulating and highly soluble proteins, such as the β-amyloid (Aβ) peptide, extracellularly deposited in AD patients brain as diffuse or compact neuritic plaques. In addition or at least to some degree, almost all of the brains of AD patients show cerebrovascular Aβ deposits, termed as cerebral amyloid angiopathy (CAA) [47,48,49]. CAA contributes to cognitive decline and dementia in AD through reduced CBF [50].

The formation of neurofibrillary tangles (NFTs) is considered a key pathological process in the etiology of AD, causing neurodegeneration and cognitive decline. The neuronal tau protein undergoes an abnormal phosphorylation contributing to typical dysfunctions of AD [51]. It has been extensively debated whether these proteins have priority over the others in the pathogenic mechanism of AD. The fact that the production of Aβ, and consequently amyloid deposition, is increased in hereditary forms of early-onset AD, points out the importance of the amyloid [52]. Furthermore, tau proteins morphology changes according to different mutations in tau genes, developing several tauopathies, among which AD is the most studied. Mutations in tau can also develop other forms of dementia, as for example frontotemporal dementia associated with Parkinsonism. In particular, in this latter pathology there is an accumulation of neurofibrillary tangles containing tau proteins, whereas there is a loss of extracellular amyloid [53].

Early-onset/genetic AD represents only a small part of total AD cases and usually affects younger people, running in families. In these cases, an increased production of Aβ peptides, that mainly compose senile plaques in the brain have been reported. On the contrary, a great amount of patients are affected with the so-called late-onset/non genetic AD (or sporadic AD) with no dysregulation in Aβ production but with a probable alteration of its clearance from the Central Nervous System (CNS) [54,55]. Moreover, Aβ peptides accumulate in perivascular space becoming toxic for the neuronal tissue. Indeed, the Aβ cascade has been so far considered the most prominent hypothesis that could explain AD pathogenesis. In physiological conditions, the amyloid precursor protein (APP) is cleaved by specific secretases into Aβ peptides. In pathological conditions, Aβ peptides aggregate in Aβ plaques depositing among neurons and promoting brain toxicity [56,57]. The Aβ cascade hypothesis points out that deposition of Aβ peptide in the brain is a central event in the pathology of AD.

Hypertension and ischemic brain injury may be a major cause of Aβ lesions in the brain. Cholinergic dysfunction may negatively affect cerebral blood flow with consequences on the normal functioning of neurovascular units [58]. Hypertension accelerates cognitive deficits, microvascular deposition of β-amyloid, vascular inflammation, blood-brain barrier (BBB) leakage and pericyte loss in TgSwDI mice [59]. Mounting evidence suggests that the chronic cerebrovascular insufficiency induced by hypertension, could be considered the initial event in the onset of dementia and not simply a by-stander effect [60]. The emerging concept is that chronic high blood pressure constantly challenges the normal function of the BBB and alters the clearance of toxic substances from the brain, like Aβ itself [54].

The BBB represents the fine crosstalk between the vascular and nervous systems [54]. The neurovascular unit defines the cellular interaction between endothelial cells of brain capillaries (forming the BBB), the astrocytic end feet, and neuronal axons. Interestingly, cholinergic terminals of neurons in the basal nucleus of Meynert directly interact with astrocytic end feet of the BBB via cholinergic receptors [54]. Therefore, the cholinergic neurons are the most affected neuronal population in AD and, together with the cerebrovascular system, represent the basic neurovascular unit for the study of the cited pathology.

Brain blood vessels are lined with brain endothelial cells (BEC), like elsewhere in the body, but in this district BEC have unique features, since they are not fenestrated and are sealed by tight junctions, which allow them to form the BBB [54]. Endothelial cells regulate the vascular tone releasing vasoactive factors, as for example NO and free radicals. Arteries and arterioles are enveloped by one or more layers of smooth muscle cells (myocytes), *i.e.*, contractile cells that regulate vascular diameter; whereas, in capillaries, myocytes are replaced by pericytes [54].

Pericytes, which belong to the vascular smooth muscle cell (VSMCs) lineage, have a common basement membrane with endothelial cells, and encircle capillaries with their cytoplasmic processes [61]. The density of pericytes processes related to capillary length suggests that they might envelope 30% to 70% of the capillary wall, with a pericyte-to-endothelia ratio in the brain of 1:3 [54]. The location of pericytes on the microvessel appears to be functionally determined. Pericytes make focal contacts with BEC through specialized junctions and, through long cytoplasmic processes, encircle the endothelial tube [62]. This close relationship with BEC allows pericytes to have many influences on brain microcirculation. Indeed, pericytes contribute to microvessel stability and cover a major part of the abluminal endothelial surface [62].

Pericytes, besides providing mechanical stability, are also able to affect vessel stability by matrix deposition, through the release and activation of signals promoting BEC differentiation and quiescence [63]. It has been reported that pericytes are contractile cells capable of regulating blood flow in brain capillaries through contraction and relaxation [64] and that they may perform the same functions as those of VSMCs in arterioles and small pial arteries [63]. It has also been hypothesized that, because of their fundamental functions, pericytes and VSMCs may play a key role in the development of dementia-related neuropathological consequences of hypertension [61,62,65]. For example, it has been shown that cerebral VSMCs isolated from AD individuals have a hypercontractile phenotype, which leads to increased arterial contractility and aberrant responses to vasoactive stimuli [66]. Moreover, in AD patients post mortem tissues and in transgenic mouse models of the pathology [66], a clear alteration of vascular related transcription factors, serum response factor (SRF) and myocardin was found.

Finally, it has been shown that vascular cells such as endothelium and pericytes, can directly affect neuronal and synaptic functions through changes in CBF, BBB integrity and clearance of toxic molecules [54].

## 4. RAGE as a Possible Molecular Mechanism for Hypertension-Induced Vascular Dysfunction and AD

The use of animal models resembling the neuropathological features of AD by Aβ overproduction, consequently resulting in its aggregation and deposition in brain parenchyma, failed to provide a dissection of the mechanisms related to vascular risk factors in the onset of late-life dementia, where a cause ascribable to the increased production of Aβ is rarely found [44,45,46]. Besides Aβ production, an important process regulating the accumulation of amyloid in the brain is represented by the balance between influx and efflux across the BBB [54]. Hence, this poses a question about the need to study the Aβ clearance mechanism at the BBB level and how vascular related factors could contribute to its dysfunction. It has been demonstrated that hypoperfusion, increased oxidative stress and impaired clearance of Aβ in the BBB determine the accumulation of neurotoxic Aβ oligomers in the brain [67]. In particular, it is well known that the exchange of Aβ between CNS, cerebrospinal fluid (CSF), and blood is an important process affecting the levels of Aβ in the brain [54]. On this issue, several studies have shown the existence of a receptor-mediated bidirectional transport of Aβ across the BBB, *i.e.*, receptor for advanced glycation end products (RAGE). RAGE mediates the influx from circulation into the brain and is present at the luminal side of the endothelium [68]. Low-density lipoprotein receptor related protein-1 (LRP-1), located at the abluminal side of the BBB, mediates instead the inverse process. Interestingly, ApoE, one of the most important risk factors associated with AD and CAA, is an LRP-1 ligand and modulates the rate of Aβ transport [69].

Interestingly, RAGE has been found implicated in several pathological states, including AD, diabetic nephropathy, and immune/inflammatory reactions of the vessel walls [70]. In addition, it has been reported that microvascular RAGE levels rise in correspondence with the onset of AD, and continue to increase linearly as a consequence of the pathologic severity of AD [71].

RAGE is able to respond to several ligands, among which the advanced glycation end products (AGEs) have been shown to be elevated in AD brains. It has been shown that AGEs can stimulate Aβ production [72] and induce tau hyperphosphorylation impairing synapse and memory [73]. RAGE is also responsible for the activation of microglia and for the production of proinflammatory mediators following Aβ binding [74]. Enhanced IL-1β and TNF-α production was observed in transgenic mice expressing human mutant APP (mAPP) in neurons and RAGE in microglia. At the same time, an increased infiltration of microglia and astrocytes and accumulation of Aβ were coupled with an accelerated deterioration of spatial learning/memory. Indeed, the blockade of microglial RAGE may have a beneficial effect for alterations in AD brains [75].

There are also recent observations describing that RAGE activation downstream the oxidative stress. In particular, it has been demonstrated that AngII-induced activation of RAGE is impaired by using free-radicals scavenger [76] and that ROS production that follows hyperglycemia provokes an increase in the expression of RAGE and its ligands [77]. It is conceivable that hypertension, known to activate vascular oxidative stress [78], could further recruit RAGE activation as an additional mechanism involved in hypertension-induced AD-like neuropathology. In fact, we demonstrated that chronic hypertension in mice determines an early up-regulation of RAGE in cerebral blood vessels [10]. More importantly, we demonstrated that genetic ablation of RAGE protected mice from hypertension-induced Aβ deposition and cognitive impairment [10]. Furthermore, AngII can up-regulate the expression of RAGE in cultured endothelial cells and increase the soluble form in the medium [79]. An inhibitor of AngII type 1 receptor is able to decrease RAGE levels in the serum of patients affected with essential hypertension, therefore suggesting a possible crosstalk between RAS and RAGE expression [79].

It is interesting to notice that RAGE has been pursued for long time as a strategy to counteract AD in patients [80]. Different inhibitors have been generated and tested in controlled clinical trials, but results have been unsatisfactory [80,81]. On this issue, we also tested one of the last RAGE specific inhibitors developed in our model of hypertension-induced AD-like pathology [10]. Interestingly, the high affinity of the tertiary amide RAGE-specific inhibitor (FPS-ZM1) [82], which blocks specific Aβ binding on RAGE V-domain , was strongly effective in protecting hypertensive mice from Aβ deposition and cognitive impairment [10,82]. Altogether, these observations demonstrate that the RAGE pathway plays a direct role in determining Aβ levels in the brain. However, it is conceivable that, in order to test the real translational potential of findings obtained in mice and to overcome the failures reported so far, it is important to find the clinical population that could benefit the most from this strategy. More recently, it has been found that, by investigating the interaction between hypertension and AD in cerebrovascular function, the administration of AngII or deoxycorticosterone acetate (DOCA)-salt to induce hypertension further aggravates the cerebrovascular effects of Aβ in transgenic TgSwDI mice with elevated brain levels of Aβ [83]. This evidence further supports the existence of mechanisms strictly entangled in the relationship between hypertension and dementia.

## 5. Conclusions

Hypertension and dementia, separately, represent two of the largest health problems worldwide in the aging population. However, when present concomitantly, they seem to potentiate the effect of each other. The structural and functional alterations induced by hypertension partly explain how the two conditions may be related at the pathophysiological level. Indeed, it is becoming increasingly clear that, besides the classical view addressing the problem of Aβ production and accumulation in the brain, the process of Aβ clearance, in absence of genetic causes, is of crucial importance in experimental settings. Basic science research on novel experimental models, which better recapitulates the additive or synergistic power of hypertension and AD, helped in elucidating the pathophysiological links between these two devastating conditions. Further studies will focus on investigating the translational potential of this link. Meanwhile, in the absence of effective therapies, evidence suggests that reaching the therapeutic target in hypertension is crucial for reducing the morbidity related not only to cardiovascular diseases, but to dementia as well.
